# Exosomal Protease Cargo as Prognostic Biomarker in Colorectal Cancer

**DOI:** 10.31557/APJCP.2021.22.3.861

**Published:** 2021-03

**Authors:** Natalia V. Yunusova, Elena A Zambalova, Marina R Patysheva, Elena S Kolegova, Sergey G Afanas’ev, Olga V Cheremisina, Alina E Grigor’eva, Svetlana N Tamkovich, Irina V Kondakova

**Affiliations:** 1 *Cancer Research Institute, Tomsk National Research Medical Center, Russian Academy of Sciences, Tomsk, Russia. *; 2 *Department of Biochemistry and Molecular Biology, Faculty of Medicine and Biology, Siberian State Medical University, Tomsk, Russia. *; 3 *Laboratory of Molecular Medicine, Institute of Chemical Biology and Fundamental Medicine, Siberian Branch of the Russian Academy of Sciences, Novosibirsk, Russia. *; 4 *Department of Clinical Biochemistry, V. Zelman Institute for the Medicine, Novosibirsk, Russia. *

**Keywords:** Plasma exosomes, CD151, metalloproteinases, 20S proteasome, colorectal cancer

## Abstract

**Objective:**

The aim of the study was to develop a model for predicting cancer risk in colorectal polyps’ patients (CPPs), as well as to reveal additional prognosis factors for Stage III colorectal cancer based on differences in subpopulations of tetraspanins, tetraspanin-associated and tetraspanin-non-associated proteases in blood plasma exosomes of CPPs and colorectal cancer patients (CRCPs).

**Methods:**

The subpopulations of CD151- and Tspan8-positive exosomes, the subpopulations of metalloproteinase at the surface of СD9-positive exosomes and the level of 20S proteasomes in plasma exosomes in 15 CPPs (tubulovillous adenomas) and 60 CRCPs were evaluated using flow cytometry and Western blotting. Logistic regression analysis was performed to predict cancer risk of CPPs.

**Results:**

The levels of 20S proteasomes in exosomes, MMP9+, MMP9+/MMP2+/EMMPRIN+ in CD9-positive blood plasma exosomes are associated with the risk of malignant transformation of colorectal tubulovillous adenomas. In patients with Stage III CRC, the levels of 20S proteasomes (less than 2 units) and MMP9+ subpopulations (more than 61%) in plasma exosomes are unfavorable prognostic factors for overall survival. The levels of 20S proteasomes and ADAM10+/ADAM17- subpopulations in CD9-positive blood plasma exosomes are the most significant values for predicting relapse-free survival.

**Conclusion:**

Protease cargo in CD9-positive blood plasma exosomes is prognostic biomarker for colorectal polyps and colorectal cancer.

## Introduction

Colorectal cancer (CRC) is the third most common cancer and the second leading cause of cancer death in men and women worldwide (Ferlay et al., 2019). Survival of patients diagnosed with CRC is significantly associated with the cancer stage. Approximately one third of patients with non-metastatic CRC develop recurrence or hematogenous metastasis within three years after diagnosis (Loree et al., 2017). Currently, there are various methods for CRC screening, including endoscopic screening and fecal occult blood testing. However, these methods have some limitations: endoscopic examinations are invasive, expensive and inconvenient, and there is an associated risk of bowel perforation, while fecal occult blood testing is non-invasive, but may not be sensitive enough to serve as an effective screening option for CRC (Garborg et al., 2013; Duffy et al., 2014; Brenner et al., 2014; Dickinson et al., 2015).

CRC typically develops from focal changes in precancerous polyps. Up to 70% of CRCs coming from adenomatous polyps. The risk of malignant transformation of hyperplastic polyps is observed in about 1% of cases. Tubular adenomas are more likely to become cancerous in 10% and tubulovillous adenomas in 23-50% of cases. Unfavorable factors associated with increased risk of CP malignant transformation include: multiple polyps (20% of them undergo malignant transformation), age (in patients aged 60 years and older, the risk of colorectal polyps (CPs) malignant transformation reaches 6-8%), tumor size (adenomas with1-2 cm in diameter undergo malignant transformation in 20-50% of cases). According to current recommendations, all CPs with the exception of inflammatory CPs should be removed. Despite the fact that polypectomy can reduce the incidence of CRC by 20%, it does not guarantee the absence of CP recurrence and cancer development. Moreover, patients diagnosed with colon adenomas were almost 6 times more likely to develop CRC than the general population (Garborg et al., 2013; Duffy et al., 2014; Brenner et al., 2014; Dickinson et al., 2015). Serum biomarkers such as CEA (cancer-embryonic antigen) and CA19-9 (carbohydrate antigen 19-9) are considered to be the best prognostic markers for CRC (Dickinson et al., 2015). However, the low sensitivity and specificity rates of these markers affects their use as biomarkers for early diagnosis, and their expression level is evaluated only for post-resection monitoring of CRC patients (Brenner at al., 2014; Wada et al., 2017).

Recent studies have shown that potential biomarkers previously found in blood plasma and urine are concentrated in exosomes (van Niel et al., 2018). Exosomes are nanosized (30–100 nm) vesicular structures that belong to a large population of extracellular vesicles constitutively released by cells to ensure intercellular communication (Colombo et al., 2014; Zhang et al., 2015). Exosomes contain a complex biomolecular cargo, which includes proteins, peptides, lipids and nucleic acids, which are able to transfer to recipient cells. It has been shown that tumor growth, angiogenesis, extracellular matrix remodeling, metastasis and immune surveillance are stimulated by exosomes (Hong at al., 2012). Tetraspanins account for 12% of the total protein composition of exosomes, and CD9, CD63, CD81 are exosomal biomarkers. Tetraspanin CD151 is associated with laminin-binding integrins and is involved in cell migration through activation of Ras, Rac1, Cdc42 (Kalluri, 2016). The involvement of CD151 in the regulation of cell motility is due to its effect on proteases. So, in association with MMP14, it regulates the sheddase activity of ADAM10 and ADAM17. It was found that overexpression of CD151 in tumor tissue correlates with low survival of cancer patients (Andreu, 2014; Romanska et al., 2015; Detchokul et al., 2014). The expression of Tspan8 correlates with the metastatic potential of carcinoma of the liver, colon and pancreas (Detchokul et al., 2014; Yue et al., 2017). About 32% of the protein composition of exosomes are enzymes. Proteases, enzymes of the hydrolase class, play an important role in the functional activity of extracellular vesicles. Tetraspanin-associated proteases include ADAM10, ADAM17, and matrix metalloproteinases (MMPs), tetraspanin-non-associated exosomal protease is 20S proteasome, PAPP-A etc. (Yunusova et al., 2018; Kondakova et al., 2020). ADAMs are multifunctional proteins that perform shedding that leads to the cleavage of the extracellular domain of transmembrane proteins (EGFR1, Her2, TGFbeta-IIIR, L1CAM, CD44, EpCAM) thereby regulating cell adhesion, migration, and intercellular interactions (Burbano et al., 2015). There is no evidence of the presence of pro- or mature forms of soluble MMPs in multivesicular bodies, but proteomics have shown the presence secreted-type (soluble) MMPs (MMP-1, -13, -2,-9, -3, -10, -7, -19) in EVs derived from various cell type and isolated from various biological fluids (blood plasma, malignant ascites, urine, saliva, synovial fluid). The significance of the transfer of MMPs, their inducer EMMPRIN and their tissue inhibitors by extracellular vesicles has not been fully established. There is an opinion that MMPs concentrated on the EV surface can degrade the extracellular matrix more efficiently, which facilitates local invasion of tumor cells, stimulates neoangiogenesis and tumor metastasis (Buzas et al., 2018; Shimoda and Khokha, 2017).

The aim of the study was to develop a model for predicting cancer risk in colorectal polyps patients (CPPs), as well as to reveal additional prognosis factors for stage III colorectal cancer based on differences in subpopulations of tetraspanins, tetraspanin-associated and tetraspanin-non-associated proteases in blood plasma exosomes of CPPs and colorectal cancer patients (CRCPs).

## Materials and Methods

*Patients*

Blood samples from CPPs (tubulovillous adenomas) (n = 15) and CRCPs with Stage T2-4N0-2M0 (n=60, 58.6±1.6 years) were obtained from the Cancer Research Institute of Tomsk National Research Medical Center (Tomsk, Russia). The comparison group consisted of 15 patients (6 men and 9 women) with colorectal tubulovillous adenomas who underwent videocolonoscopy. Colorectal cancer and other cancers were not detected in these patients. Exclusion criteria for the CRCPs group were: multiple primary CRC, Ia stage CRC (T1N0M0), mid-rectal and low-rectal cancer. The study was approved by the Local Ethics Committee of the Cancer Research Institute of Tomsk National Research Medical Center (Tomsk, Russia) (Head of the committee – Marina V. Vusik, Ph.D., MD). All patients were fully informed of the purpose and nature of the treatment and provided an informed written consent. Human samples were obtained according to the principles expressed in the Declaration of Helsinki. All patients with Stage II and III CRC underwent radical surgery (hemicolonectomy or colon resection). Adjuvant chemotherapy was given as indicated. The final stage of the disease was established after surgery in accordance with the international TNM Classification of Malignant Tumors (8^th^ Edition). 

*Exosome isolation*

Blood plasma exosomes were isolated using ultrafiltration with ultracentrifugation (Yunusova et al., 2019). Exosome samples were aliquoted and stored either at −80°C or in liquid nitrogen. The aliquots were thawed once before use. To evaluate protein concentrations, 7.5 μL of an exosome suspension was mixed with 2.5 μL of lysis buffer (0.25 M Tris-HCl, 8 % SDS, 0.2 М DTT, pH 6.8), incubated on ice (10 min), boiled (95°C for 10 min) and cooled. After centrifugation (12,000g, 10 min), the protein concentration was measured using a fluorometric protein assay (NanoOrange^®^ Protein Quantitation Kit, Molecular Probes, USA) according to manufacturer’s instructions.

*Transmission electron microscopy*

For negative staining, 10 μL of isolated exosomes samples were adsorbed for 1 min on copper grids covered with carbonized formvar film. Then the grids were exposed for 5–10 s on a drop a 2% phosphoric-tungstic acid. Grids were studied using Jem 1400 transmission electron microscope (Jeol, Japan), the images were obtained with a digital camera Veleta (Olympus Corporation, Japan).

*Flow cytometry*

Analysis of CD9/CD63/CD81/CD24 subpopulations in plasma exosomes. The 4 μm-diameter aldehyde/sulphate latex beads (Thermo Fisher Scientific, USA) were incubated with anti-CD9 (ab134375, Abcam) antibodies at room temperature for 14 h at gentle agitation. The aliquots of exosomes (about 30 μg vesicular protein) were incubated with antibody-coated latex beads in 150 μL of PBS at 4 °C for 14 h at gentle agitation. The reaction was blocked with 0.2 M glycine for 30 min at 4 °C. The exosomes-antibody-bead complexes were washed twice with washing buffer (2% EVs depleted bovine serum in PBS), were incubated with a blocking immunoglobulin G (BD Biosciences, USA) at room temperature for 10 min with washing. Then there was incubation with FITC-conjugated anti-tetraspanins (CD63, CD81) and anti-CD24 antibodies (BD Biosciences, USA) at 4°C for 50 min. The complexes were washed twice with washing buffer. Single beads were gated, and acquired in a Cytoflex (Becman Coulter, USA). Data were analyzed with CytExpert 2 Software. The median fluorescence intensity (MFI) of the sEVs was analyzed in comparison with the isotypic control (BD bioscience, USA).

Analysis of ADAM10/ADAM17 subpopulations in plasma exosomes. The analysis was performed similarly to the procedure presented above. For analysis of vesicular ADAM10/ADAM17 latex beads-antiCD9 antibody-vesicles complexes stained with anti-ADAM10 (CD156c)-PE (5 μL on test, N352704, Biolegend, USA), anti-ADAM17/TACE antibody (dilution 1:10, LSC329200/109998, LifeSpan BioSciences, USA) for 20 min at room temperature. Then complexes were stained with anti-Rabbit IgG secondary antibody, Alexa Fluor 488 (dilution 1:3000, Thermo Fisher Scientific, USA). 

Analysis of MMP9/MMP2/EMMPRIN subpopulations in plasma exosomes. The analysis was performed similarly to the procedure presented above. Latex beads-antiCD9 antibody-vesicles complexes stained with anti-EMMPRIN (CD147)-APC (5 μL on test, MAB5047, Abnova, USA), anti-MMP2-PE (0.3–0.5 μg on test, AAA444-575, Antibodies-online, Germany) and anti-MMP9-FITC (1 μg on test, AA1-708, Antibodies-online, Germany) for 20 min at room temperature.

Analysis of Tspan8/CD151 subpopulations in plasma exosomes. Evaluation of the Tspan8/CD151 exosome tetraspanin subpopulations was performed similarly to the method described above. Antibodies anti-Tspan8-PE Antibody (3 μl per test, ABIN4895321Antibodies-online, Germany), anti-CD151-APC Antibody (3 μl per test, 350405 Biolegend, USA) were used.

*Determination of the expression level of the sum of alpha and beta subunits of 20S proteasomes in exosomes*

Aliquots of exosomes (30 μl, 7 μg of exosomal protein) were incubated for 90 min on ice with 7 μl of lysis buffer (125 mM Tris- HCl, pH 7-8, 750 mM NaCl, 0.5% SDS, 5% Triton X-100) and 3 μl of protease inhibitors cocktail (1.3 mM Aprotinin (Sigma, USA), 0.33 mM Pepstatin A (ICN, USA), 1 μg/ml Leupeptin (ICN, USA)). Then, the samples were incubated with sample buffer at 95°C for 7 min and centrifuged at 13,000 g for 5 min. After centrifugation samples were applied to a 13% PAA gel for SDS-PAGE electrophoresis according to Lemmli. After electrophoresis, proteins were transferred to the PVDF membrane (Immobylon, Millipore, USA). The membrane was blocked with 1X iBind Solution (Invitrogen, USA) and incubated with primary antibody to 20S proteasomes (Anti-Proteasome 20S alpha+beta antibody ab22673, Abcam, UK, dilution 1:2,000) at 4^o^C, then , the membrane was washed and incubated with secondary antibody (goat anti-rabbit IgG-HRP, Santa Cruz Biotechnology, 1:5,000 dilution) in accordance with the instruction with automated Western blotting device iBind Western Device (Thermo Scientific, USA) for 3 hours. Further the membrane was incubated with the Amersham ECL western blotting detection analysis system (Amersham, USA). The visualization was performed on the system ChemiDoc Touch (Bio-Rad, USA). The density of the bands was estimated using “ImageLab” computer program. The results were standardized taking into account the level of CD63 in exosomes and were expressed as a related units of the level of the target protein in the exosomes in the CPPs.

*Assessment of overall and relapse-free survival*

Survival time was calculated from the start of treatment. The patients were followed up every three months for the first year and then every six months. A follow-up examination included: physical examination, chest x-ray in 2 projections, ultrasound examination of the abdominal organs, magnetic resonance imaging with intravenous contrast, computed tomography with intravenous contrast and video colonoscopy.

*Statistical analysis*

Statistical analysis was carried out using Statistica 10 (IBM SPSS Statistics 20) software. All data were expressed as medians with interquartile ranges or as means with standard errors. Mann-Whitney or Kruskal-Wallis tests were used to evaluate statistical differences between groups, where P-values < 0.05 were considered statistically significant. Logistic regression analysis was performed to predict cancer risk of CPPs. To assess the predictive performance of clinical and molecular parameters, Receiver Operating Characteristic (ROC) curves were used. Cumulative survival curves were constructed using the Kaplan–Meier method; the significance of differences in survival between groups was estimated using the Gehan-Wilcoxon test.

## Results

*Patients characteristics*

Clinical and histopathological parameters of the CRCPs are presented in Table 1.

*Characterization of blood plasma exosomes*

**Figure 1:**
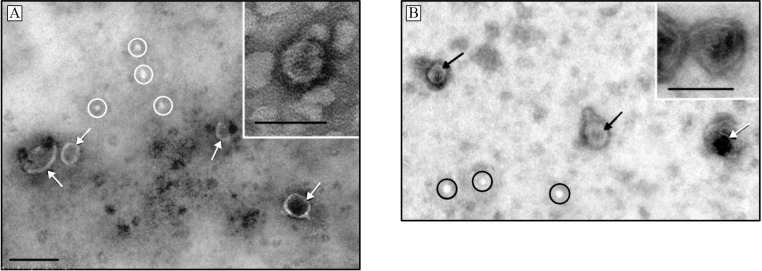
Electron Microscopic Images of Exosomes Isolated from: A – blood plasma of CPPs, B - blood plasma of CRCPs. Ovals indicate ‘non-vesicles’, arrows - exosomes. Scale bars correspond to 100 nm. Electron microscopy, negative staining

Transmission electron microscopy revealed clearly structured cup-shaped particles of low electron density with a preserved membrane in exosome preparations isolated from the blood plasma of CPPs and CRCPs (Figure 1). 

**Figure 2:**
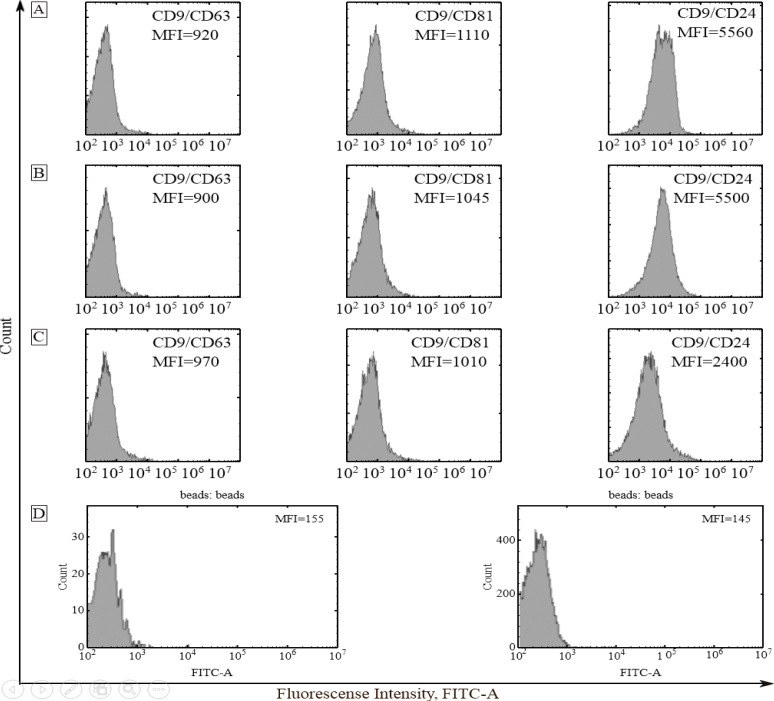
Expression of CD63, CD81 and CD24 on CD9-Positive Plasma Exosomes of CPPs (A), Stage II CRCPs (B), Stage III CRCPs (C). Isotype control and negative control (latex beads labeled anti-CD9 with anti CD81 FITC antibody) (D). Mean MFI are shown

Exosomes absorbed to aldehyde sulfate latex beads using anti-CD9 antibodies were stained with FITC-labeled antibodies to tetraspanin family receptors CD63 and CD81, as well as to CD24 receptor. Evaluation of the expression of exosome membrane proteins by a combination of conjugated and unconjugated antibodies allows the identification of various subpopulations of exosomes. To reduce the median fluorescence intensity (MFI), exosome subpopulations were distributed as follows: CD9/CD24>CD9/CD81>CD9/CD63 (plasma exosomes of CPPs); CD9/CD24>CD9/CD81>CD9/CD63 (plasma exosomes of CRCPs). In CPPs and CRCPs, populations of CD9/CD63 and CD9/CD81 were equally represented. Thus, CPPs and CRCPs had similar subpopulation composition of blood plasma exosomes (Figure 2). 

*Subpopulations of tetraspanins and proteases level in exosomes from plasma of CPPs and CRCPs*

**Figure 3:**
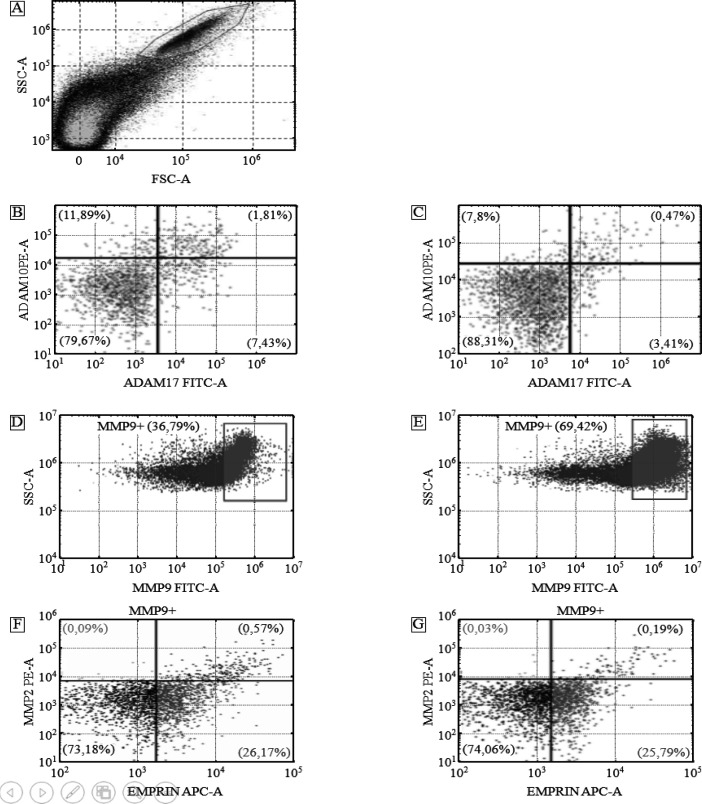
Flow Cytometry Analysis of Exosome Subpopulations. Forward scatter area (FSC-A) versus side scatter area (SSC-A) dot plot representing exosome samples adsorbed on aldehyde/sulphate latex beads labeled anti-CD9 antibody (A). Double labeling ADAM10 versus ADAM17 of blood plasma of CPPs (B) and CRCPs (C). MMP9-positive plasma exosomes population in CPPs (D) and CRCPs (E). Triple labeling MMP2 versus EMMPRIN of plasma MMP9-positive (MMP9+) exosomes of CPPs (F) and CRCPs (G)

Tspan8/CD151 subpopulations on CD9-positive exosomes isolated from plasma of CPPs and CRCPs appeared to be quite similar. Tspan8-/CD151-subpopulation predominated in both groups (CRCPs - 95.7 ± 0.66% and CPPs - 91.9 ±1.31%, respectively, p >0.05) compared to other exosome subpopulations. The ADAM10-/ADAM17- subpopulations were more frequently observed in both groups (CPPs – 76.9±3.85% and CRCPs – 88.0±4.4%, respectively, p>0.05) compared to other exosome subpopulations. Statistically significant differences in the level of ADAM10+/ADAM17- exosomes between CPPs and CRCPs were found (12.1±0.6% and 7.0±0.35%, respectively, p <0.05) (Figure 3 A, B, C). Since MMP9 was most often expressed in exosomes of CRCPs and CPPs, the emphasis was placed on the study of subpopulations of CD9+/MMP9+ exosomes (Table 2). It was revealed that MMP9-positive exosomes in blood plasma were more common in CRCPs, while triple-positive exosomes expressing MMP9 and MMP2 as well as EMMPRIN were more common in CPPs. The population of MMP9+/MMP2-/EMMPRIN- exosomes dominated (up to 73%) in both CPPs and CRCPs (3 A, D, E, F). 

**Figure 4:**
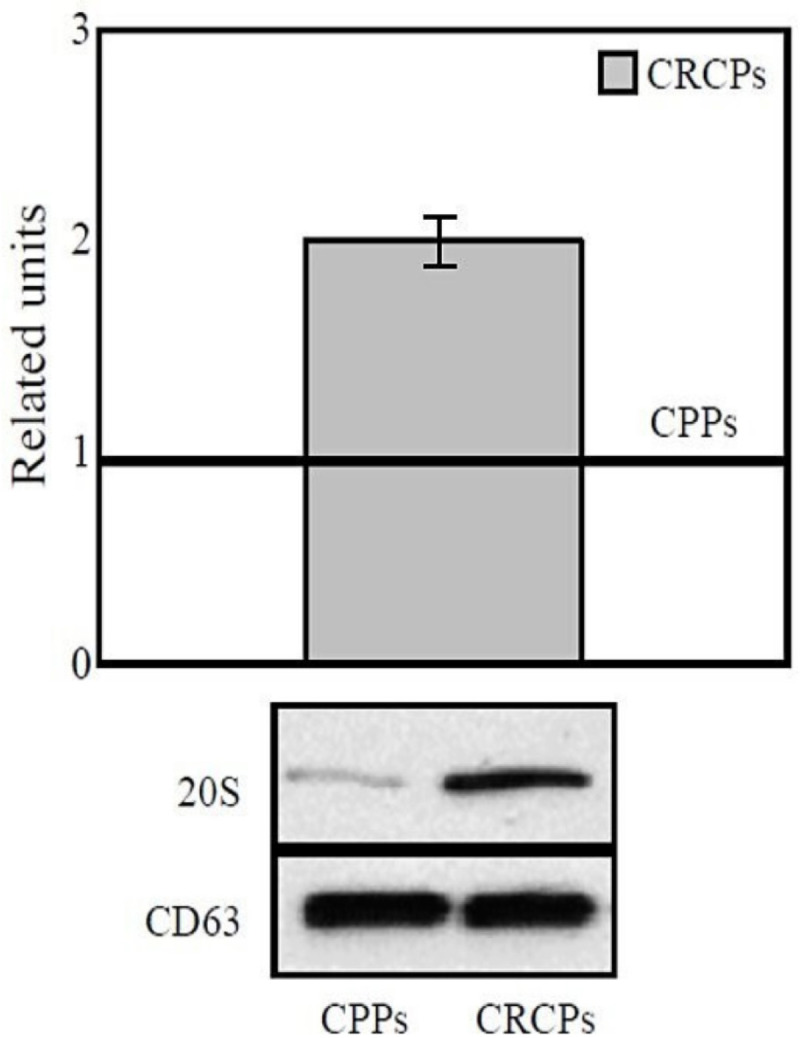
20S Proteasomes Level in Plasma Exosomes of CRCPs Compared to CPPs. The results were standardized taking into account the level of CD63 in exosomes and were expressed as a related units of the level of the target protein in the exosomes in the CPPs. Western blot analysis of plasma exosomes

Western blot analysis showed that 20S proteasomes level in exosomes from plasma of CRCPs is 1.8 times higher than the level in CPPs exosomes (Fig.4). Thus, for the first time, differences in the levels of 20S proteasomes, ADAM10+/ADAM17-, MMP9+ and MMP9+/MMP2+/EMMPRIN+ in plasma exosomes subpopulations between CPPs and CRCPs were revealed, thereby indicating the feasibility of using them to predict cancer risk.

*The exosomal tetraspanins expression and proteases level for assessment of colorectal cancer risk of CCPs*

We used a logistic regression method to identify markers of colorectal cancer risk in CPPs. The following parameters were included in the model: age, 20S proteasome level, tetraspanin subpopulations (Tspan8, CD151), the level of proteases (ADAM10, ADAM17, MMP2, MMP9) and EMMPRIN in exosomes. The model included 75 patients (CPPs – 15, CRCPs - 60). The model was significant (Χ^2^ = 55,753, p <0,001), and the quality of the model fitting was good (the Hosmer-Lemeshow test p > 0.05, Nagelkerke R-square > 0.5). It was revealed that the potential cancer risk predictors are age, 20S proteasome level in exosomes, MMP9 + and MMP9+/MMP2+/EMMPRIN+ subpopulations of CD9-positive exosomes. By regression equation was classified correctly 88.5% of patients. The sensitivity and specificity of the model is high at 93.8% and 80.0%, respectively. 

Based on the results obtained, the model of logistic regression was the following: 

F = 0,142[X_1_] + 2,481[X_2_] + 0,125[X_3_] - 4,705[X_4_] + 2,253, 

where X_1_ is the age of the patients (years), X_2_ is the level of 20S proteasomes in blood plasma exosomes (in relative units), X_3_ is the subpopulation of MMP9 + in blood plasma CD9-positive exosomes (%), X_4_ is the subpopulation of MMP9+/MMP2+/EMMPRIN + in blood plasma CD9-positive exosomes (%).

**Figure 5.**
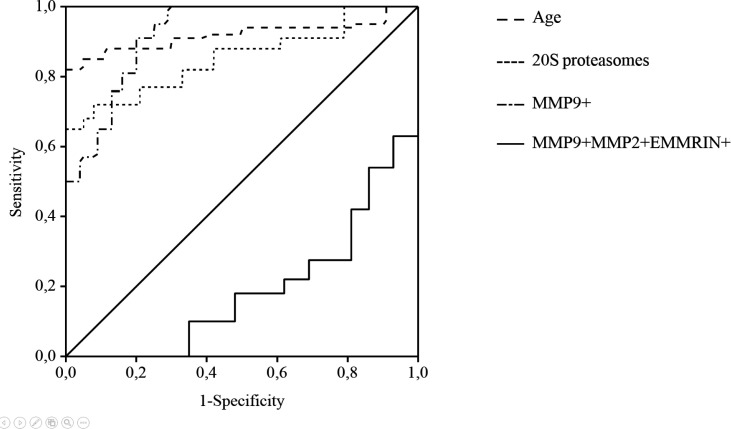
Receiver Operating Characteristics (ROC) Curve Analysis for the Prediction of Cancer Risk in CPPs

The probability value of cancer risk was calculated taking into account the value of the regression function (F) and the basis of the natural logarithm (e): 

$P=\frac{1}{1+e^{-F}}$

where P is the probability of cancer risk, e = 2.718 - the base of the natural logarithm, F is the value of the regression function. Cancer risk was assumed to be high at P-value of ≥ 0.5 and to be low at P -value of 0<0.5. For discriminating between the malignant disease group and the comparison group, an optimal cut-off point was indicated at 1.83 related units for 20S proteasome level (sensitivity=92%, specificity=80%, AUC=0.850, P<0.003), at 61% for MMP9+ (sensitivity=88%, specificity=80%, AUC=0.925, P<0.001) and at 0.28% for MMP9+/MMP2+/EMMPRIN+ (sensitivity=90%, specificity=80%, AUC=0.812, P<0.008) subpopulations, at 62 years for age of patients (sensitivity=95%, specificity=80%, AUC = 0.913, P < 0.001). ROC curves for discriminating between the CRCPs and CPPs is shown in Figure 5.

*Exosomal protein cargo as an additional prognostic factor for colorectal cancer at III stage*

**Figure 6:**
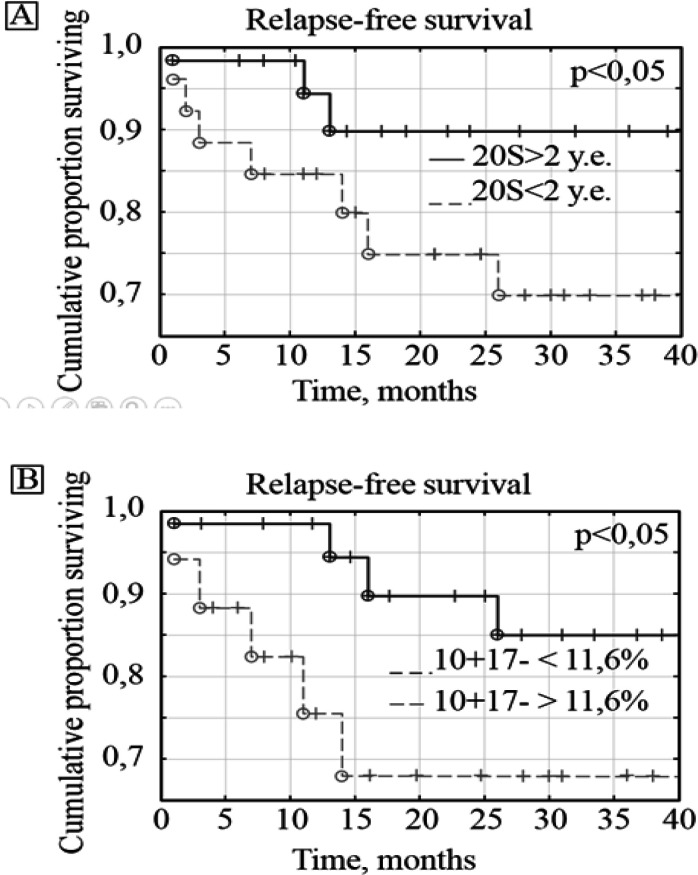
Relapse-Free Survival of CRCPs Depending on the Level of 20S Proteasomes (A) and ADAM10+/ADAM17- (B) in Blood Plasma Exosomes

Sixty patients with stage II - III CRC were followed up for 2 years. Since patients with stage II CRC had no disease progression, additional prognostic factors associated with 20S proteasome levels and subpopulations of tetraspanins and proteases were identified only in patients with stage III CRCPs. Among 30 patients with stage III CRC, 23 (76%) were alive and 7 (24%) died. Disease recurrence occurred in 9 (32%) patients. The 20S proteasome and MMP9+ expression levels were identified as prognostic factors for the overall survival. The most significant values for assessing the relapse-free survival of patients were the levels of 20S proteasomes and ADAM10+/ADAM17- subpopulations of CD9-positive blood plasma exosomes (Figure 6).

**Таble 1:** Clinical and Histopathological Parameters of the CRCPs

		N (%)
Sex	Male	29 (49)
	Female	31 (51)
Age	≤59	18 (30)
	>59	42 (70)
Stage	Stage II	30 (50)
	Stage III	30 (50)
Localization	Right colon	18 (30)
	Left colon	42 (70)
Tumor grade	G1-G2	53 (88)
	G3	7 (12)

**Table 2:** Metalloproteinase Compositions (%) at the Surface of CD9-Positive Exosomes in CPPs and CRCPs samples, M ± m

Subpopulations	CPPs	CRCPs	P-value
MMP9+	40.0±8.1	60.9±6.8	<0.05
MMP9-	60.0±7.1	39.1±6.8	<0.05
MMP9+/MMP2+/EMMPRIN+	0.57±0.12	0.28±0.05	<0.05
MMP9+/MMP2-/EMMPRIN-	72.7±6.6	71.1±2.4	>0.05
MMP9+/MMP2-/EMMPRIN+	26.8±4.00	28.6±2.00	>0.05
MMP9+/MMP2+/EMMPRIN-	0.03±0.01	0.03±0.01	>0.05

## Discussion

The morphology of blood plasma exosomes isolated from CRCPs was found to have no difference with that of patients with other cancers (Yunusova et al., 2019; Tugutova et al., 2019; Tamkovich et al., 2019). Moreover, we showed that the levels of 20S proteasomes in exosomes, MMP9+ and MMP9+/MMP2+/EMMPRIN+ exosome subpopulations in CD9-positive exosomes were significant for predicting colorectal cancer risk in CPPs. Exosomal 20S proteasomes are increased in breast and ovarian cancer patients compared with that in healthy females (Tamkovich et al., 2019). MMP9+/MMP2+/EMMPRIN+ subpopulation of CD9-positive blood plasma exosomes reduced the cancer risk in CPPs. Similar data were obtained for ovarian cancer (Yunusova et al., 2019). Subpopulation of MMP9+/MMP2+/EMMPRIN+ at the surface of blood plasma exosomes in high-volume ascites ovarian cancer patients was reduced compared to that in low-volume ascites patients. In accordance with previously published data, the ascites volume for serous high-grade carcinomas correlated both with the outcome of primary surgery and overall survival and low-volume ascites patients have a better prognosis (Yunusova et al., 2019). 

Tetraspanins are among the major proteins of exosomes. Tetraspanins act as molecular mediators by binding to a variety of proteins assembled into special enriched membrane domains. They form the so-called tetraspanin-rich microdomains that interact with a large number of transmembrane and cytosolic signaling proteins. Their main partners are tetraspanins themselves, integrins, adhesion receptors, signal receptors, matrix metalloproteinases (Yanez-Mo at al., 2009). Tspan8 and CD151 tetraspanins have been shown to contribute to tumor progression (Detchokul et al., 2014; Yue et al., 2017). However, in our study, exosomal tetraspanins were not identified as prognostic factors for patients with colorectal polyps or colorectal cancer and can be considered mainly as a microenvironment for metalloproteinases, at least for a given cancer localization. Sheddases ADAM10 and ADAM17 are structurally linked to tetraspanin domains. Tutanov et al., (2020) revealed the importance of the expression of ADAM10 at CD9-positive exosomes in the biodistribution of free plasma exosomes and blood cell-surface-associated vesicles in healthy women and in luminal breast cancer patients. The expression of ADAM10 on CD9-positive exosomes likely may be important in developing the luminal subtype of breast cancer. Secreted MMPs, other extracellular matrix proteins are known to form the exosome protein corona, and they are functionally linked to tetraspanin domains and ADAM proteases (Buzas et al., 2018, Shimoda and Khokha, 2017). 

In current study we revealed increased levels of 20S proteasomes in plasma exosomes of CRCPs compared to those in CPPs. Earlier was found that the level of 20S proteasomes in blood plasma exosomes was associated with metabolic markers and stage in CRCPs (Tugutova etal., 2019). In CRCPs, the level of 20S proteasomes in circulating exosomes was high, but the chymotrypsin-like activity was low or absent. The chymotrypsin-like activity of extracellular proteasomes is known to be restored to normal levels after purification procedures. The chymotrypsin-like activity is likely to be inhibited by an unknown protein reversibly linked to extracellular proteasomes (Kulichkova et al., 2017). We found that the levels of 20S proteasomes (less than 2 units) and MMP9+ subpopulations (more than 61%) in exosomes were unfavorable prognostic factors for the overall survival of patients with stage III CRC. The most significant values for predicting relapse-free survival were the levels of 20S proteasome in exosomes and ADAM10+/ADAM17- subpopulation in CD9-positive blood plasma exosomes of CRCPs. Thus, exosomal proteins were found to be associated with survival of cancer patients and can serve as promising biomarkers for predicting adverse outcomes. These molecules should be combined with other clinical and molecular biomarkers to evaluate the optimal treatment option for cancer patients.

## Author Contribution Statement

Author Contributions. Conceptualization – Natalia V.Yunusova, Irina V.Kondakova; analysis of data – Elena A. Zambalova., Marina R. Patysheva, Elena S. Kolegova, Alina E.Grigor’eva, Sergey G. Afanas’ev, Olga V. Cheremisina; preparation of manuscript – Natalia V.Yunusova, Elena A. Zambalova, Svetlana N.Tamkovich
